# Increased epicardial adipose tissue thickness as a sign of subclinical atherosclerosis in patients with rheumatoid arthritis and ıts relationship with disease activity ındices

**DOI:** 10.1007/s11739-024-03542-6

**Published:** 2024-04-05

**Authors:** Ayşe Bahar Keleşoğlu Dinçer, Haluk Furkan Şahan

**Affiliations:** 1 Rheumatology Department, Dışkapı Yıldırım Beyazıt Research and Training Hospital, Ankara, Altındağ Turkey; 2 Cardiology Department, Etlik Şehir Hastanesi, Ankara, Turkey

**Keywords:** Atherosclerosis, Coronary artery disease, Rheumatoid arthritis, Inflammation

## Abstract

Epicardial adipose tissue is a novel cardiometabolic risk factor and indicator of subclinical atherosclerosis. We aimed to evaluate the epicardial adipose tissue thickness in rheumatoid arthritis (RA) patients and its association with disease activity scores. A total of 81 rheumatoid arthritis patients and 70 age- and sex-matched healthy individuals were recruited for this cross-sectional study. Epicardial adipose tissue thickness (EATT) was measured by transthoracic two-dimensional echocardiography. Tender and swollen joint counts were recorded at the time of inclusion. The laboratory tests included erythrocyte sedimentation rate (ESR), C-reactive protein (CRP), rheumatoid factor, anti-citrullinated protein antibodies, and serum lipid levels. Disease activity was calculated based on Disease Activity Scores for 28 joints (DAS-28) ESR and CRP, the Simple Disease Activity Index (SDAI), and the Clinical Disease Activity Index (CDAI). Epicardial adipose tissue thickness was significantly higher in the RA patients compared to the healthy controls (*p* < 0.001). We found statistically significant correlations of EATT with all disease activity indices (*p* < 0.001) and CRP (*p* = 0.002). According to a cut-off value of 6.4 mm determined for epicardial adipose tissue thickness, the RA patients with thickness ≥ 6.4 mm had higher disease activity scores and CRP levels. In the multivariable regression analysis, only SDAI score was found as an independent risk factor for increased EATT (OR, (95%CI), 13.70 (3.88–48.43), *p* < 0.001). Epicardial adipose tissue thickness measurement by echocardiography is a reliable method for assessing subclinical atherosclerosis in rheumatoid arthritis patients, and a higher disease activity score is an independent risk factor for coronary artery disease.

**Introduction ** Rheumatoid arthritis (RA) is a chronic systemic autoimmune disease with a prevalence of 0.5–1% resulting in progressive joint damage [1]. It not only affects the synovial joints but also presents with extra-articular manifestation, and most importantly, is associated with increased morbidity and mortality [2, 3]. The leading cause of increased mortality in RA patients is the cerebrocardiovascular events [4]. Autoimmunity and systemic inflammation have the major contribution to the increased rates of adverse cardiovascular events [1, 5, 6].

The progression of atherosclerosis is multifactorial, and inflammation also has an important role [7]. It was reported that people with RA who have persistent inflammation are more likely to develop subclinical atherosclerosis [7]. In a prospective study, it was shown that failure to adequately control RA activity increases the risk of developing subclinical atherosclerosis, and cardiovascular events four to eight times [2]. Inflammation markers such as the erythrocyte sedimentation rate (ESR), C-reactive protein (CRP), tender joint counts (TJC), swollen joint counts (SJC), the presence of a deformity, and extra-articular disease manifestations have been demonstrated to be associated with an increased risk of atherosclerosis [8].

Carotid-intima-media thickness and the presence of carotid plaques have been shown as reliable predictors of subclinical atherosclerosis, and to be increased in many inflammatory rheumatic diseases including RA [3, 9–11]. Nevertheless, epicardial adipose tissue (EAT) is a novel cardiometabolic risk factor, and an indicator of atherosclerosis [12].The epicardial adipose tissue is located on the free wall of the right ventricle and the left ventricular apex between the visceral pericardium, and the myocardium surrounding the subepicardial coronary arteries [11]. It is a complex endocrine organ that secretes many proinflammatory cytokines such as interleukin-1 (IL-1), IL-6, tumor-necrosis factor, and proatherogenic factors in excessive amounts [13]. Studies have shown that EAT plays a role in the initiation and progression of coronary artery disease (CAD) due to its close anatomical location to the adventitia of coronary arteries and its paracrine and local inflammatory effects [14, 15]. Epicardial adipose tissue thickness (EATT) was demonstrated to be associated with atherosclerotic plaques, and an independent predictor of CAD [16]. Although the radiological measurement of EAT thickness provides a more accurate result, transthoracic echocardiographic (TTE) measurement is still an accurate, inexpensive, non-invasive, and easily accessible method [12].

In this study, we aimed to evaluate EAT thickness in RA patients without prior CAD and its association with disease activity indices to determine the risk of subclinical atherosclerosis in RA patients.

## Patients and methods

### Study population

Patients aged ≥ 18 years, diagnosed with RA according to the 2010 American College of Rheumatology/European League Against Rheumatism Rheumatoid Arthritis Classification Criteria, who presented to a tertiary rheumatology outpatient clinic between May 2022 and September 2022 were included in the study [17]. Pregnant or breast-feeding patients, and patients with known concurrent comorbidities like arterial hypertension, diabetes mellitus, coronary artery disease, cerebrovascular disease, chronic liver or kidney diseases, solid or hematological malignancies, acute or chronic infections, or other autoimmune/autoinflammatory diseases were excluded from the study. Healthy control (HC) group was selected from patients who admitted to the rheumatology outpatient clinic and were examined thoroughly regarding any rheumatic disease. Patients who were not diagnosed with any autoimmune and/or autoinflammatory rheumatic diseases as well as the other exclusion criteria diseases stated above according to patient history, physical examination and laboratory test results were also recruited for the study. The control group was randomly selected by stratification according to age and sex.

The study was conducted in accordance with the principles of the Declaration of Helsinki and approved by the Ethics Committee of the institution (Number:109/37, Date:19.04.2021). Written informed consent was obtained from all participants.

### Demographic and clinical characteristics

Information on the demographic and clinical characteristics of the participants was obtained at the time of recruitment. Their disease onset, smoking status, treatment characteristics, Tender joint count (TJC), swollen joint count (SJC), and patient’s and physician’s visual analogue scale assessments (0–100 cm) were recorded. The body mass index (BMI) was calculated as body weight divided by height squared (kg/m^2^). Blood samples were collected in the morning after 8 h fasting, and fasting glucose, total cholesterol, low-density lipoprotein (LDL), triglyceride (Tg), and high-density lipoprotein (HDL) levels were measured. Other laboratory tests included hepatic transaminases, serum creatinine, erythrocyte sedimentation rate (ESR), C-reactive protein (CRP), rheumatoid factor (RF), anti-citrullinated protein antibody (ACPA), and complete blood count (CBC).

### Assessment of rheumatoid arthritis disease activity

The disease activity assessments for the RA patients were performed according to disease activity scores—ESR based on 28-joints (DAS28-ESR), DAS28-CRP, the Simplified Disease Activity Index (SDAI), and the Clinical Disease Activity Index (CDAI) [18–20].

### Transthoracic echocardiography

The RA patients and healthy controls underwent detailed transthoracic two-dimensional imaging, and tissue Doppler imaging echocardiography procedures were performed with a Philips EPIQ 7 device (Philips Ultrasound; Bothel, WA, USA) by standard techniques [21, 22]. The echocardiographic examinations were performed by a cardiology specialist, and the evaluation was blinded. Routine echocardiographic measurements were obtained according to the recommendations of the American Society of Echocardiography [23]. For the epicardial adipose tissue measurements, the patient was placed in the lateral decubitus position, and optimal parasternal long-axis images were obtained. For optimization, the aortic root was determined as the reference, and the right ventricular free wall and aortic annulus were ensured to be in the midline [24, 25]. Epicardial adipose tissue is defined as an echo-free space between the outer border of the myocardial wall and the visceral layer of the pericardium. After long-axis imaging, EAT measurements were made in the mid-chordal section by short-axis imaging. The measurements were made in three cardiac cycles, their mean value was calculated, and the data included the end-diastole part, the section taken just before the R wave in the ECG [26].

### Statistical analysis

IBM SPSS Statistics for Windows, version 26.0 (SPSS Inc, Chicago, IL, USA) was used to perform the statistical analyses. The variables were investigated using visual (histograms, probability plots) and analytical (Kolmogorov–Smirnov/Shapiro–Wilk test) to determine whether they were normally distributed. Due to non-normally distribution of the continuous variables, they were presented as median and IQR values. The categorical variables are presented as frequency values. Since non-normally distribution was observed for numerical continuous variables in the intergroups comparisons, Mann–Whitney *U*-test was used and for categorical variables chi-squared or Fischer’s test were used when appropriate. Correlation analysis between epicardial adipose tissue thickness and disease activity indices was performed using Spearman’s test. In order to determine the independent risk factors affecting epicardial adipose tissue thickness, variables with *p* < 0.25 in univariate analyzes and traditional cardiovascular risk factors were taken into account. Among these variables, when the correlation coefficient between them was about 0.6, the one with the larger p value or a lower clinical significance was excluded from the study. The remaining variables were included in the logistic regression analysis by the enter method. Odds ratios (OR) and their 95% confidence intervals were calculated for the independent risk factors identified. Model accuracy was evaluated with the Hosmer–Lemeshow goodness of fit statistics. Power analyses were performed using the G* Power version 3.0.10 power and sample size calculation program. A *p*-value < 0.05 was accepted as statistically significant.

## Results

A total of 81 RA patients and 70 age- and sex-matched HC were enrolled in this case–control study. Both groups were similar in terms of their BMI values and smoking status. None of the patients or the HC were on lipid-lowering drugs and in both groups, blood lipid levels were within normal range (i.e., LDL < 160 mg/dL, Tg < 150 mg/dL, HDL > 40 mg/dL). The demographic characteristics and laboratory results of the RA patients and HC are summarized in Table 1. The RF and ACPA parameters were positive in 79% and 69% of the RA patients, respectively. Methotrexate (MTX) was the most commonly used disease-modifying anti-rheumatic drug (DMARD) at a rate of 62% among the RA patients, which was followed by leflunomide (LEF) in 27%, hydroxychloroquine sulfate (HCQ) in 54%, and biological DMARD in 6%. The majority of the patients had moderate disease activity according to their DAS28-ESR, DAS28-CRP, and SDAI scores. The epicardial adipose tissue thickness values of the RA patients were significantly higher compared to the HC (*p* < 0.001). The demographic, clinical, laboratory and treatment characteristics of RA and HC are summarized in Table 1.

**Table 1:** Demographic, clinical, and laboratory characteristics of rheumatoid arthritis and healthy control groups

	RA (*n* = 81)	HC (*n* = 70)	*p* value
Demographic characteristics			
Age (years), median (IQR)	57 (46.5–64)	53.5 (45.5–63)	0.356
Sex (Female), *n* (%)	53 (65.4)	50 (71.4)	0.430
Body mass index (kg/m^2^), median (IQR)	28.5 (23.2–31)	28.1 (24.2–32.3)	0.676
Current smoking, n (%)	21 (25.9)	20 (28.6)	0.715
Laboratory characteristics, median (IQR)			
Total cholesterol levels (mg/dL),	183 (165.8–211.8)	192 (168.8–216.8)	0.450
High-density lipoprotein levels (mg/dL)	54 (44–68)	49 (39.5–56.5)	*0.035*
Low-density lipoprotein levels (mg/dL)	115 (94.3–132)	120 (101.8–139.5)	0.386
Tryglycerid levels (mg/dL)	111 (80.5–160.8)	139 (88.8–218.8)	*0.035*
Creatinine (mg/dL)	0.69 (0.6–0.82)	0.7 (0.64–0.83)	0.669
ESR (mm/h)	11 (6.5–18)	9 (3–16)	*0.022*
CRP (mg/L)	4.33 (1.98–9.95)	2.53 (1.50–4.65)	*0.001*
Epicardial adipose tissue thickness (mm)	6.4 (5.8–7.2)	3.35 (3–3.95)	< *0.001*
Clinical features of RA patients			
Disease duration (months), median (IQR)	12.5 (3.8–72)		
Rheumatoid factor (IU/mL), median (IQR)	57.3 (17.9–140.4)		
Rheumatoid factor positivity, *n* (%)	64 (79.0)		
Anti-citrullinated protein antibody, median (IQR)	77.1 (0.5–200)		
Anti-citrullinated protein antibody positivity, *n* (%)	56 (69.1)		
DAS28-ESR, median (IQR)	3.27 (2.48–4.17)		
DAS28-CRP, median (IQR)	3.92 (3.20–4.94)		
SDAI, median (IQR)	15.9 (9.2–27.9)		
CDAI, median (IQR)	9 (5.5–18)		
Treatment features of RA patients, *n* (%)			
Corticosteroids	63 (77.8)		
Mean corticosteroid dosage, median (IQR)	5 (5–7.5)		
Methotrexate	50 (61.7)		
Leflunomid	22 (27.2)		
Sulfasalazine	4 (4.9)		
Hydroxychloroquine sulfate	44 (54.3)		
Biological drugs	5 (6.2)		
*Tofacitinib*	*4 (4.9)*		
*Tocilizumab*	*1 (1.2)*		

**Fig. 1:**
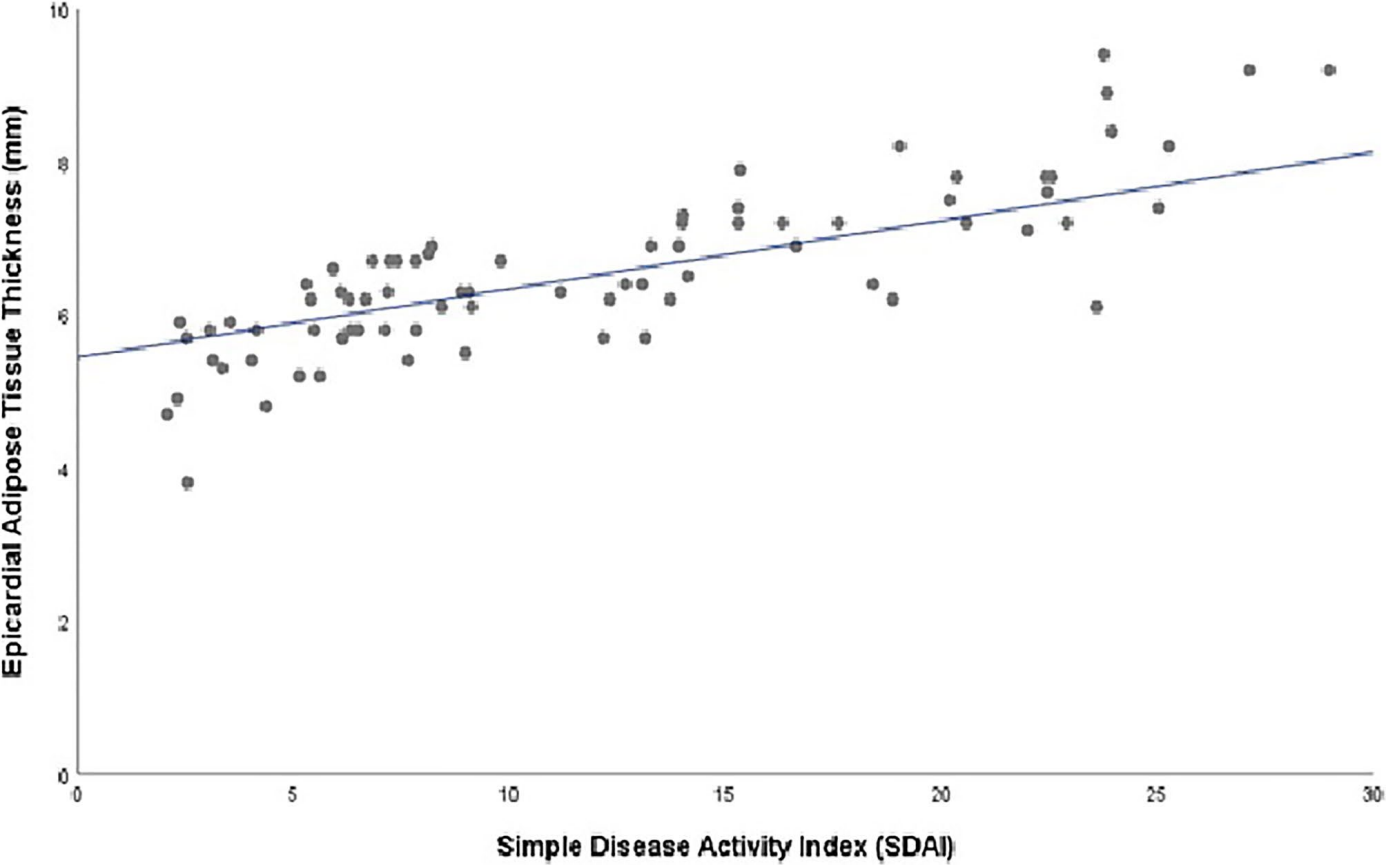
Relationship Between Epicardial Adipose Tissue Thickness and the Simple Disease Activity Index

The EATT results of the RA patients showed strong, positive and significant correlations with their DAS28-ESR (rho: 0.778, *p* < 0.001), DAS28-CRP (rho: 0.882, *p* < 0.001), SDAI (rho: 0.835, *p* < 0.001) and CDAI (rho: 0.838, *p* < 0.001) results (Fig. 1). The EATT also showed a moderate correlation with CRP (rho: 0.344, p = 0.002). On the other hand, no statistically significant correlation was found between EATT, RA disease duration, age and body mass index (*p* = 0.331, *p* = 0.435, and *p* = 0.103, respectively). Table 2 summarizes the association of EATT with disease activity indices, disease duration, age, and BMI.

**Table 2:** Correlation of epicardial adipose tissue thickness with acute phase reactants, rheumatoid arthritis disease activity indices

	EATT	
	rho	*p*-value
DAS28-ESR	0.778	< *0.001*
DAS28-CRP	0.822	< *0.001*
SDAI	0.835	< *0.001*
CDAI	0.838	< *0.001*
ESR (mm/h)	0.075	0.506
CRP (mg/L)	0.344	*0.002*
Disease duration (months)	-0.111	0.331
Age (years)	0.088	0.435
BMI (kg/m^2^)	0.187	0.103

*BMI* Body mass index, *EATT* Epicardial adipose tissue thickness, *DAS28-ESR* 28-Joint Disease Activity Score with Erythrocyte Sedimentation Rate, *DAS28-CRP* 28-Joint Disease Activity Score with C-Reactive Protein, *SDAI* Simple Disease Activity Index, *CDAI* Clinical Disease Activity Index, *CRP* C-reactive protein

The RA patients were divided into two groups as the remission and low disease activity group and the moderate-to-high disease activity group based on their disease activity index values (DAS28-ESR, DAS-28-CRP, SDAI, and CDAI). The EATT was found significantly higher in the moderate-to-high disease activity group compared to the remission-low disease activity group in all four disease activity indices (Table 3).

**Table 3:** Epicardial Adipose Tissue Thickness in Rheumatoid Arthritis Patients with Remission-to-Low Disease Activity and Moderate-to-High Disease Activity

	EATT (mm), median (IQR)	*p*-value
DAS28-ESR		
remission-low disease activity (*n* = 39)	5.8 (5.4–6.3)	< *0.001*
moderate-high disease activity (*n* = 42)	7.2 (6.4–7.95)	
DAS28-CRP		
remission-low disease activity (*n* = 20)	5.7 (5.2–5.88)	< *0.001*
moderate-high disease activity (*n* = 61)	6.8 (6.2–7.55)	
SDAI		
remission-low disease activity (*n* = 41)	5.8 (5.45–6.3)	< *0.001*
moderate-high disease activity (*n* = 40)	7.2 (6.43–8.05)	
CDAI		
remission-low disease activity (*n* = 41)	5.8 (5.45–6.3)	< *0.001*
moderate-high disease activity (*n* = 40)	7.2 (6.43–8.05)	

*EATT* Epicardial adipose tissue thickness, *DAS28-ESR* 28-Joint Disease Activity Score with Erythrocyte Sedimentation Rate, *DAS28-CRP* 28-Joint Disease Activity Score with C-Reactive Protein, *SDAI* Simple Disease Activity Index, *CDAI* Clinical Disease Activity Index, *CRP* C-reactive protein

The median EATT among the RA patients was found as 6.4 mm. When this value was used as the cut-off value, the EATT ≥ 6.4 was accepted as ‘increased EATT’. The RA patients were then sub-grouped as patients with EATT < 6.4 (*n* = 39) and those with EATT ≥ 6.4 (*n* = 42). The median age, sex, disease duration, BMI, smoking status, and seropositivity results were not significantly different between the groups (*p* = 0.526, p = 0.246, *p* = 0.285, *p* = 0.087, *p* = 0.573, *p* = 0.823, and *p* = 0.434, respectively). Nevertheless, CRP levels were significantly higher in the patients with EATT ≥ 6.4 compared to the patients with EATT < 6.4 (*p* = 0.007). Likewise, the disease activity scores of the RA patients with increased EATT were significantly higher in comparison to the RA patients with EATT < 6.4 (Table 4).

**Table 4:** Comparison of Demographic, Clinical, Laboratory, and Treatment Characteristics of Rheumatoid Arthritis Patients with and without Increased Epicardial Adipose Tissue Thickness

	EATT < 6.4 mm (*n* = 39)	EATT ≥ 6.4 mm (*n* = 42)	*p*-value
Demographic features			
Age (years), median (IQR)	53 (46–64)	57 (47–63.3)	0.526
Sex (female) *n* (%)	28 (71.8)	25 (59.5)	0.246
Disease duration (months), median (IQR)	18 (8–81)	12.5 (2.3–60)	0.285
BMI (kg/m^2^), median (IQR)	27.3 (22.1–29.7)	29.3 (24.2–33.3)	0.087
BMI > 30(kg/m^2^), *n* (%)	8 (21.6)	13 (32.5)	0.284
Current smoking, *n* (%)	9 (23.1)	12 (28.6)	0.573
Laboratory features, median (IQR)			
Total cholesterol levels (mg/dL),	175 (156–199.5)	188 (174–219)	0.088
High-density lipoprotein levels (mg/dL)	54.5 (4.53–65)	52.5 (42.8–69.3)	0.745
Low-density lipoprotein levels (mg/dL)	107 (85.8–130.5)	120.5 (104.3–132.8)	*0.040*
Triglycerides (mg/dL)	105.5 (77–137.5)	119.5 (93–168)	0.140
ESR (mm/h)	11 (4–19)	11.5 (7.8–18)	0.535
CRP (mg/L)	3.3 (1.2–6.07)	6.27 (2.99–10.33)	*0.007*
Rheumatoid factor positivity, *n* (%)	30 (78.9)	34 (81)	0.823
Anti-citrullinated protein antibody positivity, *n* (%)	25 (65.8)	31 (73.8)	0.434
Disease Activity Scores, median (IQR)			
DAS28-ESR	2.48 (1.95–3.17)	4.07 (3.27–4.56)	< *0.001*
DAS28-CRP	3.23 (2.54–3.66)	4.83 (4.05–5.26)	< *0.001*
SDAI	6.3 (4–9)	17.1 (13–23.1)	< *0.001*
CDAI	6 (4–8)	16.5 (11–22)	< *0.001*
Treatment Characteristics, *n* (%)			
Corticosteroids	32 (82.1)	31 (73.8)	0.373
Corticosteroid mean dosage, median (IQR)	5 (2.5–6.9)	5 (5–10)	0.326
Methotrexate	23 (59)	27 (64.3)	0.623
Leflunomide	14 (35.9)	8 (19)	0.088
Biological drugs	1 (2.6)	4 (9.5)	0.361

*EATT* Epicardial adipose tissue thickness, *BMI* Body mass index, *DAS28-ESR* 28-Joint Disease Activity Score with Erythrocyte Sedimentation Rate, *DAS28-CRP* 28-Joint Disease Activity Score with C-Reactive Protein, *SDAI* Simple Disease Activity Index, *CDAI* Clinical Disease Activity Index, *ESR* Erythrocyte sedimentation rate, *CRP* C-reactive protein

We analyzed the independent variables that had the greatest impact on higher EATT (i.e.; EATT ≥ 6.4) further using logistic regression models. The components with a *p*-value < 0.100 and the components of metabolic syndrome were included in the analysis. In the multivariate analysis, SDAI was found to be an independent risk factor for increased EATT (*p* < 0.001) (Table 5).

**Table 5:** Univariable and Multivariable Regression Analysis of Parameters Associated with Increased Epicardial Adipose Tissue Thickness

	Univariate analysis		Multivariate analysis	
	OR (95% CI)	*p*-value	OR (95% CI)	*p*-value
Age (1 unit increase)	1.01 (0.98–1.05)	0.455	1.00 (0.95–1.05)	0.957
Sex (female)	0.58 (0.23–1.47)	0.248	1.10 (0.29–4.23)	0.890
Body Mass Index (1 unit increase)	1.06 (0.98–1.16)	0.154	1.06 (0.93–1.19)	0.415
Low-density lipoprotein ≥ 100 mg/dL	3.37 (1.18–9.63)	*0.024*	1.85 (0.44–7.86)	0.404
Triglyceride ≥ 150 mg/dL	1.99 (0.69–5.75)	0.201	1.29 (0.30–5.45)	0.732
Simple Disease Activity Index (SDAI) (moderate-high disease activity)	16.76 (5.57–50.40)	< *0.001*	12.67 (3.84–41.82)	< *0.001*

## Discussion

This study demonstrated that EATT was increased in the RA patients compared to the healthy controls as a predictor of subclinical atherosclerosis. Additionally, EATT was correlated with the disease activity scores of the patients with RA that include DAS28-ESR, DAS28-CRP, SDAI, and CDAI. This study also showed that only the increased disease activity score was an independent risk factor for increased EATT in RA patients. To the best of our knowledge, although, there are a few studies in the literature evaluating EATT in RA patients with contradictory results about its association with disease activity, this is the first study that included SDAI and CDAI as novel disease activity markers of RA and found a significant relationship between disease activity and increased EATT.

Epicardial adipose tissue is a true visceral adipose tissue surrounding the subepicardial coronary arteries [24]. It is a metabolically active paracrine and endocrine organ secreting many proinflammatory and proatherogenic cytokines, chemokines, and adipokines [27]. In addition to systemic effects, due to its direct contact with coronary arteries, it may also directly contribute to coronary atherogenesis [28]. Accordingly, EAT plays a role in the development of subclinical atherosclerosis through vascular inflammation and endothelial dysfunction [29]. Several studies have revealed the association of EAT with adverse cardiovascular events ranging from asymptomatic to overt coronary artery disease independently of other risk factors [30]. In recent years, it was shown that the pathogenesis of atherosclerosis is a dynamic process in which inflammation plays a role in all stages [7]. This study showed that EATT increases in patients with RA, which is a chronic inflammatory disease, as a predictor of subclinical atherosclerosis. Likewise, in the studies conducted by Temiz et al. and Fatma et al., EATT was significantly higher in RA patients in comparison to HC [31, 32]. Lima-Martinez et al. conducted a study with 34 female RA patients and showed that healthy women had the lowest EATT values compared to RA patients, and RA patients receiving biological DMARDs had lower EATT in comparison to patients receiving non-biological DMARDs [33]. In the study by Başpınar et al., the epicardial fat area measured by computed tomography was significantly higher in RA patients in comparison to healthy individuals [34]. On the other hand, in the study by Ormseth et al., EATT was not significantly higher in RA patients in comparison to HC (*p* = 0.06), although RA patients with metabolic syndrome (*n* = 56) had significantly higher EATT values compared to RA patients without metabolic syndrome (*n* = 101) [35]. There are a few studies in the literature that have evaluated EATT in other rheumatic diseases in which increased EATT values have been demonstrated in patients with systemic sclerosis, systemic lupus erythematosus (SLE), ankylosing spondylitis, and inflammatory bowel disease. [36–39]

Corticosteroids have major effects on adipose tissue metabolism via glucocorticosteroid specific receptors which are at the highest density in the visceral adipose tissue. It has been shown that high doses of steroid increase the visceral adipose tissue [40]. Besides, the long-term steroid use is associated with weight gain, even obesity, which in return causes metabolic syndrome, and are related with the features of metabolic syndrome. Kitterer et al., performed the first study that evaluated the effect of long-term steroid therapy on epicardial and pericardial fat deposition. Their study included 61 patients with various rheumatic diseases (mainly vasculitis), and age-sex-BMI matched healthy controls. Larger epicardial fat was found in patients treated with steroids in comparison to steroid-naϊve patients. In addition, significantly higher epicardial fat was found in patients on high-dose steroids (defined as > 7.5 mg prednisone equivalent per day for at least 6 months) compared to patients on low-dose streoids (ie., < 7.5 mg prednisone equivalent per day for at least 6 months). However, no significant difference was observed in epicardial and pericardial adipose fat between patients on low-dose steroids and steroid-naϊve [40]. In the cross-sectional study that included 162 SLE patients and 86 healthy controls, EAT was significantly higher in SLE patients than in controls, and when adjusted for age, sex, race, and waist circumference, only cumulative corticosteroid dose was remained significantly associated with the EAT volume [37]. Even though, corticosteroid use may have had an influence on higher EATT in RA patients in comparison to healthy controls in our study, the majority of our patients were on low-dose steroid (median 5 mg, [IQR, 5–7.5]). Likewise, in the study of Karpouzas et al., 139 RA patients who underwent coronary angiography for EAT measurement and coronary plaque assessment, the EAT volume was found higher in patients with atherosclerosis compared to patients without atherosclerosis, and it was related with the number of segments with plaque as well as mixed plaque presence in RA patients. Yet, they demonstrated that prednisone use did not alter the association between the EAT volume and the plaque burden [41].

Moreover, we showed that EATT was significantly correlated with disease activity scores, and in the logistic regression analysis, only disease activity was demonstrated to be an independent risk factor for increased EATT. Besides EAT’s contribution to systemic inflammation, systemic inflammation in return causes adipogenesis leading to an accumulation of fat in epicardial tissue promoting a positive feed-back on inflammation and accelerated atherosclerosis [28, 41]. Therefore, an increase in EATT is expected to be seen in RA patients with higher disease activity scores for which we found a cut-off value of 6.4 mm, and patients with EATT ≥ 6.4 mm in our study were found to have higher DAS28-ESR, DAS28-CRP, SDAI, and CDAI scores. Data about the correlation of EATT with disease activity in rheumatic diseases are insufficient and conflicting. Similar to our study, in the study by Alpaydın et al., DAS28 scores were demonstrated to be positively correlated with EATT (*r* = 0.365, *p* = 0.02) [42]. In a study conducted with 115 psoriasis patients, higher EATT values were found in the patients in comparison to healthy individuals, and EATT was correlated with psoriasis disease activity scores [43]. Psoriasis Area and Severity Index scores were also shown as an independent predictor of EAT [43]. Again, in a study conducted with systemic sclerosis patients (*n* = 30), EATT was demonstrated to be correlated only with disease activity scores (*r* = 0.45, *p* = 0.01) [44]. Contrary to our results, Petra et al. and Ormseth et al. did not find any significant correlations between ESR, CRP, DAS28-CRP scores, and EATT values [35, 45]. Temiz et al. showed significant correlations of EATT with ESR, CRP, and Health Assessment Questionnaire scores, but EATT was not correlated with DAS28 scores [31]. Nonetheless, we believe that, as the strength of our study, we showed the correlation of EATT not only with DAS28 but also with other novel disease activity scores of RA, namely SDAI and CDAI.

Another noteworthy finding of our study was that in the univariate and multivariate regression analyses, we found SDAI as an only predictor of increased EATT, and therefore, an independent risk factor for subclinical atherosclerosis in RA patients. As far as we know, this is the first study that showed higher disease activity as a risk factor for EATT. In our study, we did not observe any correlation between EATT and disease duration. Similar results were also found in other studies which reported that the progression of the disease does not result in a significant increase in EATT [31, 45]. These results may be interpreted as disease activity, rather than disease duration, has a greater influence on increased risk of cardiovascular events, even in the early stages of the disease.

There is copious amount of data regarding the association of EATT with coronary atherosclerosis, yet, the number of prospective large-population based studies on the prognostic value of increased EATT for the prediction of future major adverse cardiac events (MACE) are limited. In a prospective study in which 200 patients with a clinical diagnosis of CAD who underwent left and right coronary angiography were followed-up for 26 months, the incidence of death due to cardiovascular events was significantly higher in patients with EATT > 7 mm [46]. The Heinz-Nixdorf Recall study is a prospective population cohort that evaluated the predicted value of EAT volume for coronary events. Of the 4,093 participants without a baseline coronary heart disease, during a follow-up year of 8.0 ± 1.5 years, 130 of them had fatal or non-fatal coronary events developed. The EAT volume was found higher in subjects who developed any coronary event (121 mL vs 95 mL, *p* < 0.001). The study also showed that the incident of coronary events increased with the EAT volume, and the subjects in the highest EAT volume quartile had a fivefold higher risk of having a coronary event in comparison to subjects in the lowest EAT volume quartile [47]. Again, in a recent study that included 2068 asymptomatic participants without a prior coronary artery disease from the prospective, randomized EISNER (Early Identification of Subclinical Atherosclerosis by Noninvasive Imaging Research), MACE were reported in 223 (11%) of participants during a mean follow-up of 13.9 ± 3 years. By using deep-learning which is a subset of machine-learning, the EAT volume and attenuation were quantified. The EAT volume was shown to be significantly higher in participants with MACE than without MACE (90.6 [IQR, 67.4–128.7] cm^3^ vs. 77.0 [54.7–103.3] cm^3^, *p* < 0.001). In multivariate analysis, adjusted after atherosclerotic cardiovascular disease risk score, the EAT volume was found to be associated with increased risk of MACE (HR, 1.35 [95% CI, 1.07–1.68], *p* = 0.009). Again, there was a strong correlation between the EAT volume and the subsequent myocardial infarction and cardiac mortality (HR, 1.49 [95% CI, 1.00–2.21], *p* = 0.046) [48]. On the contrary, Albuquerque et al. evaluated 194 patients with a known coronary artery disease who entered a phase II cardiac rehabilitation program for a mean follow-up of 3.6 ± 1.3 years, and they showed that the EAT when measured by echocardiography was not a predictor of MACE (HR, 1.32 [95% CI, 0.75–2.31, *p* = 0.33]); which was defined as diagnosis of acute coronary syndrome, coronary revascularization, stroke, ventricular arrhythmias requiring hospitalization and death of any cause [49]. However, this study had some limitations such as shorter follow-up period when compared to other studies, smaller study population and the presence of higher comorbid diseases of the patients. Furthermore, as the study population included patients with a known previous coronary artery disease, this study may be limited to explain the role of EATT for subclinical atherosclerosis and the future cardiovascular events.

The main limitations of our study were its cross-sectional design and the lack of a measurement of arterial blood pressure and waist circumference. However, as we excluded patients with other cardiovascular risk factors such as diabetes mellitus, hypertension, and hyperlipidemia, we could make a more accurate analysis of predictors influencing EATT. Another limitation of this study may be the use of two-dimensional echocardiography to measure EATT as its sensitivity may be lower especially in obese patients and that two-dimensional measurement may not fully assess the entirety of EAT. Yet, a good correlation of echocardiographic EAT with magnetic resonance imaging measurements was previously shown [50]. Echocardiography is still an easily accessible and inexpensive method for measuring EATT. Again, all echocardiographic measurements in this study were performed by a single cardiologist, which might be another limitation of this study. The last limitation of our study was the lack of assessing plaque burden and plaque types in coronary arteries as well as different arterial sites by imaging methods such as computed tomography angiography or vascular ultrasound which could have improved our results of the association of EATT with subclinical atherosclerosis.

## Conclusions

The epicardial adipose tissue thickness is an emerging cardiometabolic risk factor, and its measurement by echocardiography is a reliable and an easy method for predicting subclinical atherosclerosis in RA patients. Due to its correlation with disease activity and the finding that a higher disease activity score is an independent risk factor for higher EATT, the tight control of disease activity in RA reduces not only the risk of joint damage and disability but also the risk of adverse cardiovascular events and mortality in RA patients. Larger, prospective and mechanistic studies are needed to elucidate the association between EATT and atherosclerosis in patients with rheumatoid arthritis.

## Data Availability

The data that support the findings of this study are available from the corresponding author upon reasonable request.
